# Systemic and Cerebral Vascular Endothelial Growth Factor Levels Increase in Murine Cerebral Malaria along with Increased Calpain and Caspase Activity and Can be Reduced by Erythropoietin Treatment

**DOI:** 10.3389/fimmu.2014.00291

**Published:** 2014-06-19

**Authors:** Casper Hempel, Nils Hoyer, Anna Kildemoes, Charlotte Bille Jendresen, Jørgen Anders Lindholm Kurtzhals

**Affiliations:** ^1^Centre for Medical Parasitology, Department of Clinical Microbiology, Copenhagen University Hospital, Copenhagen, Denmark; ^2^Department of International Health, Immunology and Microbiology, University of Copenhagen, Copenhagen, Denmark

**Keywords:** cerebral malaria, erythropoietin, neuropathology, VEGF, hypoxia, brain, inflammation

## Abstract

The pathogenesis of cerebral malaria (CM) includes compromised microvascular perfusion, increased inflammation, cytoadhesion, and endothelial activation. These events cause blood–brain barrier disruption and neuropathology and associations with the vascular endothelial growth factor (VEGF) signaling pathway have been shown. We studied this pathway in mice infected with *Plasmodium berghei* ANKA causing murine CM with or without the use of erythropoietin (EPO) as adjunct therapy. ELISA and western blotting was used for quantification of VEGF and relevant proteins in brain and plasma. CM increased levels of VEGF in brain and plasma and decreased plasma levels of soluble VEGF receptor 2. EPO treatment normalized VEGF receptor 2 levels and reduced brain VEGF levels. Hypoxia-inducible factor (HIF)-1α was significantly upregulated whereas cerebral HIF-2α and EPO levels remained unchanged. Furthermore, we noticed increased caspase-3 and calpain activity in terminally ill mice, as measured by protease-specific cleavage of α-spectrin and p35. In conclusion, we detected increased cerebral and systemic VEGF as well as HIF-1α, which in the brain were reduced to normal in EPO-treated mice. Also caspase and calpain activity was reduced markedly in EPO-treated mice.

## Introduction

Cerebral malaria (CM) is one of the most severe complications of malaria causing substantial morbidity and mortality mainly in Sub-Saharan Africa (1). At present, the pathogenesis remains incompletely understood but includes cytoadhering, infected erythrocytes, leukocytes, and platelets as well as dysregulated inflammation and coagulation cascades (2). Due to the apparent cerebral hypoxia in human and murine CM (3–6), adjunctive strategies, which aim to overcome this, could potentially improve outcome.

The physiological response to hypoxia is stabilization of hypoxia-inducible factor (HIF)-1α and HIF-2α, which will dimerize with the β subunit and via binding to hypoxia responsive elements adapt the cell to low oxygen levels (7). HIF-1α and HIF-2α upregulate the transcription of a multitude of cytokines and growth factors (8, 9), but the two transcription factors induce expression of different proteins (10). One of the HIF-regulated proteins is the pleiotropic cytokine erythropoietin (EPO) mainly regulated by HIF-2α (11). EPO has been associated with protection of cells and tissue beyond the hematopoietic lineage (12, 13). Furthermore, it improves survival in murine CM (5, 14–16) and is a safe adjunctive treatment in Malian children (17). Also, the cerebral hypoxia detectable in terminally ill CM mice was reversed by exogenous EPO treatment (5).

Besides EPO, the expression of vascular endothelial growth factor-A (VEGF) is HIF-dependently upregulated in response to hypoxia (18), mainly by HIF-1α (19). VEGF is a survival factor for the endothelium but also stimulates opening of the blood–brain barrier (BBB) to facilitate angiogenesis and tissue oxygenation (20). This event is essential in developmental angiogenesis when organs are vascularized. Thus, hypoxia may stimulate unwanted BBB disruption during CM and pathological angiogenesis (21). Increased levels of VEGF and its cleaved receptors (VEGFR1/Flt-1 and VEGFR2/Flk-1) have been found in plasma and brains from CM patients (22–27) suggesting an association with cerebral pathology. On the other hand, high levels of EPO have been associated with a lower risk of neurological sequelae in children suggesting a neuroprotective effect (23).

*Plasmodium berghei* ANKA was recently shown to induce high VEGF levels in plasma promoting acute lung injury in mice (28). Despite noticeable cerebral hypoxia in murine CM using *P. berghei* ANKA (5), it is not known how hypoxia affects angiogenic signaling in murine CM. Here we assess the hypoxia-associated transcription factors and proteins in the brain in terminally ill CM mice as well as the plasma levels of angiogenesis-associated proteins.

## Materials and Methods

### Mice, parasites, and infection

Forty, 7 weeks old, female C57BL/6 mice (Taconic, Ejby, Denmark) were used for the experiment. The mice were randomly assigned to one of four experimental groups: uninfected and saline-treated (UninfSal), uninfected and EPO-treated (5000 IU/kg, day 4–7 post infection (p.i.)) (UninfEPO), infected with 10^4^ *P. berghei* ANKA and saline-treated day 4–7 p.i. (InfSal) or infected with 10^4^ *P. berghei* ANKA and EPO-treated day 4–7 p.i. (InfEPO). Injections of parasites and treatments were performed intraperitoneally (i.p.) and the volume was in all cases 200 μl. Cryo-preserved parasites were passed once in C57BL/6 mice counting viable parasites only for the experimental infection, as previously described (14). Uninfected mice received isotonic saline, which was used as diluent for the parasite inocula. EPO was also diluted in saline and thus vehicle-treated mice received saline only. Parasitemia was determined using flow cytometry by staining circulating cells with acridine orange as previously described (29). Body temperature was measured with a rectal probe (Ellab, Denmark) during the infection. A drop below 32°C was used as a proxy for death as previously described (30, 31). Animal experiments were approved by the Danish Animal Inspectorate (license number 2006/561-1128).

### Terminating the animal experiment and tissue processing

At day 8, InfSal mice showed clinical signs of CM including decreased body temperature, impaired movement, convulsions, and loss of coordination and all mice were killed. Behavioral changes were assessed qualitatively by placing mice on a 1 cm thin bar and by placing the mice on the cage lid and gradually increasing steepness. In deep anesthesia (mixture of 63 μg fentanyl, 2 mg fluanisone, and 1 mg midazolam pr. mouse), blood was collected in heparin from the orbital sinus and plasma was separated by centrifugation and stored at −20°C until use. The mice were transcardially perfused with heparinized (15000 IU/l, Leo Pharma, Denmark) isotonic saline and brain tissue was removed, snap-frozen in liquid nitrogen, and stored at −20°C until used.

### Western blots

Frozen brains were thawed on ice and homogenized in ice cold lysis buffer [50 mM Tris–HCl, 5 mM EDTA, 1% Triton X-100, 1 mM dithiothreitol (DTT)] with added protease inhibitors (Complete Mini, Roche, Denmark) using a Heidolph disperser (SilentCrusher M, Heidolph Instruments, Germany). Protein content of the lysates was determined using the Lowry assay (DC protein assay, Bio-Rad, CA, USA). The proteins were reduced in Laemmli buffer with 200 μM DTT (Sigma-Aldrich, Copenhagen, Denmark) and boiled for 5 min before separation on 10% polyacrylamide gels (BisTris, Life Technologies, Carlsbad, CA, USA) at 150 V (constant V, EPS2A200, Amersham Biosciences, Ge Healthcare, Brondby, Denmark) for 60–70 min using MES or MOPS buffer (Life Technologies) depending on protein size (Table 1). Thirty micrograms protein was loaded into each well. Proteins were transferred to polyvinylidene membranes (Immun-Blot, Bio-Rad) at 30 V (constant V, Amersham Biosciences) for 60 min. Membranes were blocked with 5% skimmed milk powder (Fluka, Sigma-Aldrich) or 5% bovine serum albumin (Sigma-Aldrich) in tris-buffered saline (TBS, Sigma-Aldrich) depending on the protein being detected (Table 1) for 60 min at room temperature. Proteins were detected with primary antibodies diluted in blocking solution (Table 1) over night at 4°C. After washing in TBS supplemented with 0.05% Tween-20 (Sigma-Aldrich), horse radish peroxidase (HRP)-conjugated secondary antibodies (Dako, Glostrup, Denmark) diluted in blocking solution were applied for 60 min at room temperature. Antibody binding was visualized with chemiluminescent substrates: Super Signal or West Femto (Pierce, Thermo-Fischer Scientific, IL, USA) on a gel-doc imager (Bio-Rad XRS, Bio-Rad) depending on abundance. Expression levels were normalized to β-tubulin levels (Abcam, UK).

**Table 1:** **Overview of antibodies used for western blots**.

Antibody	Company	Catalogue number	Dilution for WB (×)	Blocking agent	Running buffer	Luminescent substrate
HIF-1α	Novus Biologicals	NB100-131A1	2000	Skim milk	MOPS	Femtosignal
HIF-2α	Thermo Scientific	PA1-16510	500	Skim milk	MOPS	Femtosignal
Erythropoietin	Santa Cruz	Sc-7956	250	Skim milk	MES	ECL Plus
α-Spectrin	Millipore	MAB1622	1000	Skim milk	MOPS	Femtosignal
P35/25	Cell Signaling Technology	C64B10	1000	BSA	MES	ECL Plus
β-tubulin	Abcam	Ab6046	2000	Depending on target	Depending on target	Depending on target

*Antibodies were diluted in 5% skimmed milk powder in TBS (FLUKA) for WB and in 5% BSA in TBS (Sigma-Aldrich). Depending on size of the protein of interest, samples were separated with either a MES or MOPS based running buffer (Life Technologies). Depending on antigen abundance, the antibody recognition was assessed with either ECL plus or femtosignal enhancement (Pierce)*.

### ELISA

Plasma was analyzed for VEGF, soluble Flt-1 (sFlt-1), and soluble Flk-1 (sFlk-1) according to manufacturer’s instructions (Quantikine, R&D Systems, UK). Angiopoietin-1 levels were measured in 25–50 μl plasma according to manufacturer’s instructions with the modification that blocking was achieved with 5% BSA (Quantikine DuoSet, R&D Systems). Brain homogenates (diluted to 2 mg protein/ml) were analyzed for VEGF content according to manufacturer’s instructions (Quantikine, R&D Systems). Brain VEGF levels were adjusted to pg VEGF/mg total protein.

### Statistical analyses

When data followed a normal distribution and had similar variances they were analyzed by one-way ANOVA followed by *post hoc t*-tests with Holm correction. When data did not display normal distribution and equal variance, Kruskal–Wallis test with pair-wise Wilcox tests with Holm correction was applied. These data are shown as median with interquartile ranges in box plots. All statistics was carried out using R for windows version 2.15.2 (32).

## Results

### Clinical parameters

**Figure 1:**
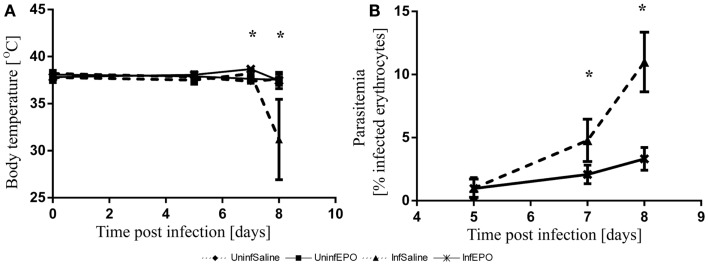
**Progression of body temperature and parasitemia during the course of infection**. **(A)** Body temperature remained stable until day 6 p.i. and increased slightly in infected mice. InfSal mice displayed clinical signs of CM at day 8 p.i. and had significantly lowered body temperature (*p* < 0.005). **(B)** Parasitemia rose gradually in both groups but did not increase as fast in InfEPO mice from day 7 p.i and onward (*p* < 0.05). Data are represented as mean values and error bars display standard deviation. Significant deviations from uninfected, saline-treated mice are denoted with an asterisk.

The infection progressed in a similar manner to what has been described before (5, 33). At day 8 p.i. the majority of the InfSal mice were terminally ill with clinical signs of murine CM. Ninety percentage had impaired movement and coordination. Thirty percentage had convulsions. InfEPO mice showed only minor clinical signs of CM (only 20% had ruffled fur). Statistical analyses revealed a significant change in body temperature on day 7 p.i. (Figure 1A, *p* < 0.001) and day 8 p.i. (*p* < 0.001), but not earlier (*p* > 0.10). At day 7 p.i., InfEPO mice had significantly higher body temperature than both groups of uninfected mice (*p* < 0.003) and InfSal mice had significantly higher body temperature than UninfSal mice (*p* = 0.008). At day 8 p.i., InfSal mice had significantly lower body temperature than any other group of mice (*p* < 0.005). Parasitemia rose gradually to similar levels in both infected groups until day 5 (Figure 1B, *p* = 0.8). From day 7 p.i. and onward, InfSal mice had significantly increased parasitemia compared with InfEPO (day 7 p.i.: *p* = 0.002; day 8 p.i.: *p* < 0.001).

### Increased cerebral levels of VEGF, EPO and their transcription factors in terminal CM

**Figure 2:**
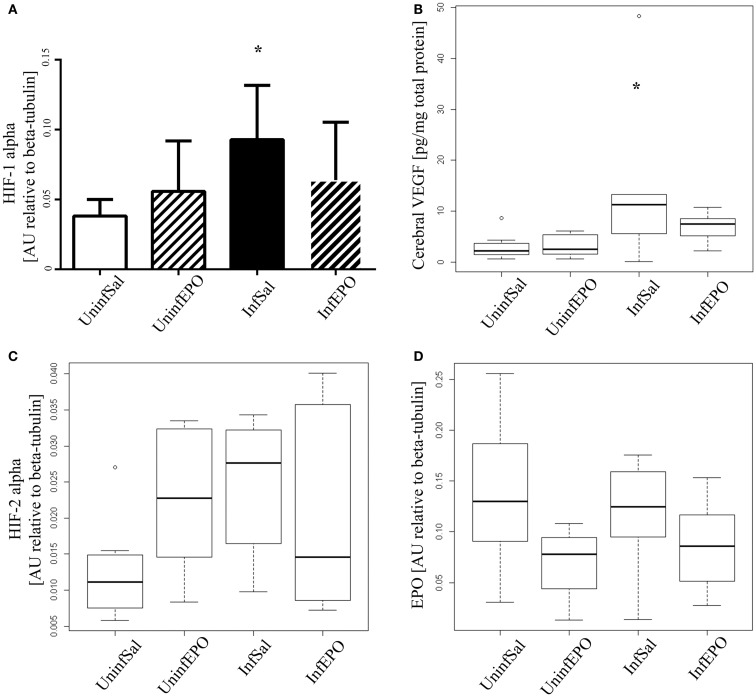
**Cerebral expression of HIF-1α, VEGF, HIF-2α, and EPO**. **(A)** HIF-1α levels were significantly increased in InfSal mice compared with UninfSal (*p* = 0.04). The other groups were statistically indistinguishable. **(B)** VEGF was analyzed with ELISA, showing a significant increase in InfSal mice compared with uninfected mice (*p* = 0.02). InfEPO was increased though not statistically significantly. **(C)** HIF-2α expression was similar in all four groups. **(D)** Also, EPO was expressed in the same level in all four groups. Bar charts **(A)** show mean values and standard deviation. Box plots **(B–D)** show median values and interquartile ranges. Whiskers show Tukey hinges; open circles are outliers. Asterisks denote significant deviations from uninfected, saline-treated mice.

From western blotting, we detected a significant increase of HIF-1α in terminal CM (Figure 2A, *p* = 0.04). Only InfSal mice were significantly different from UninfSal mice (*p* = 0.04).

Correspondingly, we detected a change in cerebral VEGF levels (Figure 2B, *p* = 0.008). In terminally ill CM mice, cerebral VEGF content was increased significantly compared to UninfSal mice (*p* = 0.02). Other groups were statistically indistinguishable from UninfSal mice. In contrast to HIF-1α, the main transcriptional regulator of EPO, cerebral HIF-2α, was expressed at comparable levels in all four experimental groups (Figure 2C, *p* = 0.1). Similarly, cerebral EPO levels were comparable in all groups (Figure 2D, *p* = 0.2).

### Calpain and caspase-3 activity in terminal CM

**Figure 3:**
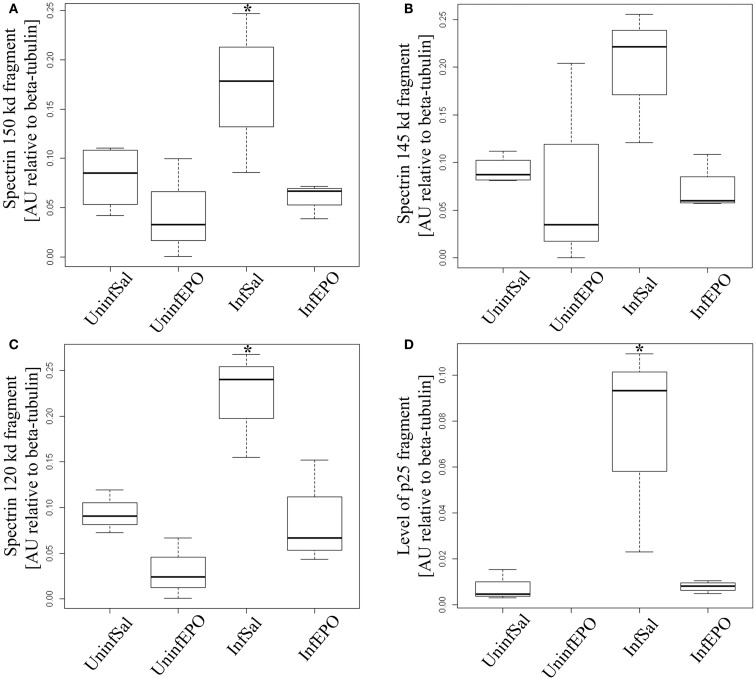
**Calpain and caspase activity in terminal CM**. Activity of caspase-3 and calpain was assessed by looking for specific cleavage products. **(A)** Analyzing the 150 kDa fragment of α-spectrin specific for calpain activity show a significant increase in InfSal mice (*p* = 0.04) compared with uninfected mice. **(B)** No significant change was noted when analyzing the 145 kDa fragment, also specific for calpain activity. **(C)** The 120 kDa band specific for caspase-3 activity was markedly increased in InfSal mice (*p* < 0.01). **(D)** p35 is cleaved by calpain activity into a smaller fragment, p25. This fragment was not detectable in UninfEPO mice, but was significantly increased in InfSal mice (*p* < 0.05). Box plots show median values and interquartile ranges. Whiskers show Tukey hinges; open circles are outliers. Asterisks denote significant deviations from uninfected, saline-treated mice.

We used non-erythroid α-spectrin as a marker of protease activity, since it contains distinct cleavage sites for calpain- and caspase-3 activity (34). α-Spectrin is cleaved by calpain, resulting in additional bands at 150 and 145 kDa, and by caspase-3 activity (apoptosis pathway) resulting in a band at 120 kDa. While total α-spectrin levels remained unaltered (results not shown), one band associated with calpain activity was significantly upregulated in InfSal mice (150 kDa, Figure 3A, *p* = 0.04) while the other was not (145 kDa, Figure 3B, *p* = 0.07) compared with UninfSal mice. Also caspase-3 activity was significantly increased (Figure 3C, *p* = 0.001) in InfSal mice as compared to the other groups (*p* < 0.01).

The activation of calpain activity was confirmed by assessing p35 levels, since calpain activity results in cleavage of p35 into a smaller protein at 25 kDa (p25) (34). p35 levels were indistinguishable between groups (*p* = 0.2, data not shown), while p25 levels were significantly different (Figure 3D, *p* = 0.02). InfSal mice had significantly higher p25 levels than other groups (*p* < 0.05) and p25 levels were not detectable above background in UninfEPO mice.

### Increased plasma levels of VEGF and decreased soluble Flk-1 levels in terminal CM

**Figure 4:**
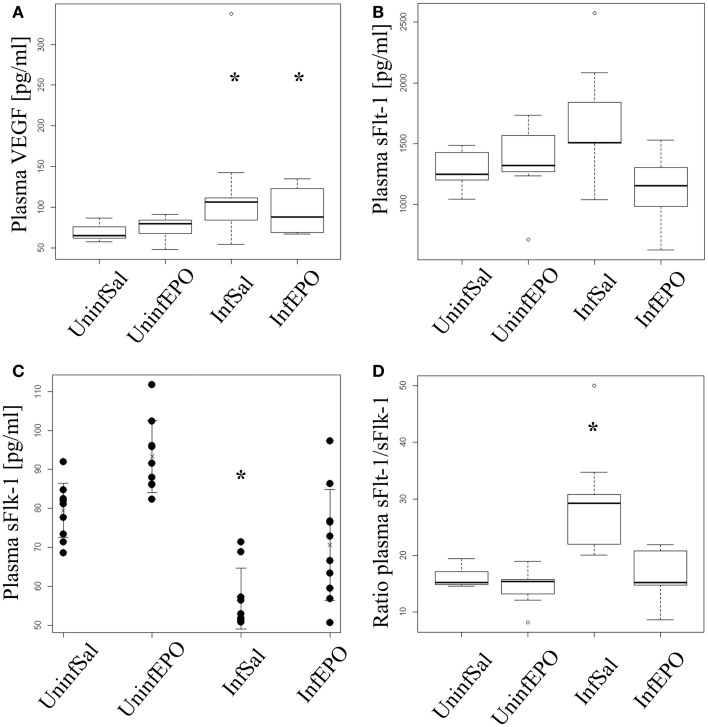
**Plasma levels of VEGF, soluble Flt-1 and soluble Flk-1**. Plasma was extracted from euthanized mice at day 8 p.i. and analyzed by ELISA. **(A)** VEGF levels increased significantly due to infection (*p* < 0.05). Both infection groups had significantly increased plasma levels of VEGF (*p* = 0.04 saline-treated, *p* = 0.03 EPO-treated). **(B)** Soluble Flt-11 was largely unaffected by infection and treatment though significant changes were noted (*p* = 0.02). No groups deviated from UninfSal mice (*p* > 0.14). **(C)** Soluble Flk-1 was significantly affected by both EPO treatment and infection. Soluble Flk-1 levels were significantly decreased due to CM (*p* < 0.001). On the contrary, EPO treatment led to an increased level of this receptor in both infected (*p* = 0.03) and uninfected mice (*p* = 0.02) compared with the corresponding saline-treated control groups. Yet, infection strongly decreased sFlk-1 levels in EPO-treated mice (*p* < 0.001) compared with UninfEPO mice. **(D)** When taking the ratio of sFlt-1 to sFlk-1 only InfSal was significantly different from uninfected mice (*p*<0.002). Box plots **(A,B,D)** show median values and interquartile ranges. Whiskers show Tukey hinges; open circles are outliers. Strip chart **(C)** show the mean value as a cross and whiskers represent standard deviation. Each dot represents the plasma level in one mouse. Asterisks denote significant deviations from uninfected, saline-treated mice.

Infection lead to a significant increase in plasma VEGF levels (*p* = 0.004, Figure 4A). Both InfSal and InfEPO mice had significantly higher levels compared with uninfected controls (*p* = 0.04 and *p* = 0.03, respectively). sFlt-1 was changed at day 8 p.i. (*p* = 0.02, Figure 4B). However, only InfEPO-treated vs. InfSal were statistically distinguishable (*p* = 0.04). sFlk-1 levels were significantly decreased in terminal murine CM (*p* < 0.001, Figure 4C). EPO treatment led to an increased level of this receptor in both infected (*p* = 0.03) and uninfected mice (*p* = 0.02) compared with saline-treated control groups. Yet, infection still decreased sFlk-1 levels in InfEPO mice (*p* < 0.001) compared with UninfEPO mice. The sFlt-1/sFlk-1 ratio was higher in InfSal than any other group (*p* < 0.002, Figure 4D).

### Plasma angiopoietin-1 levels are decreased in terminally ill mice

**Figure 5:**
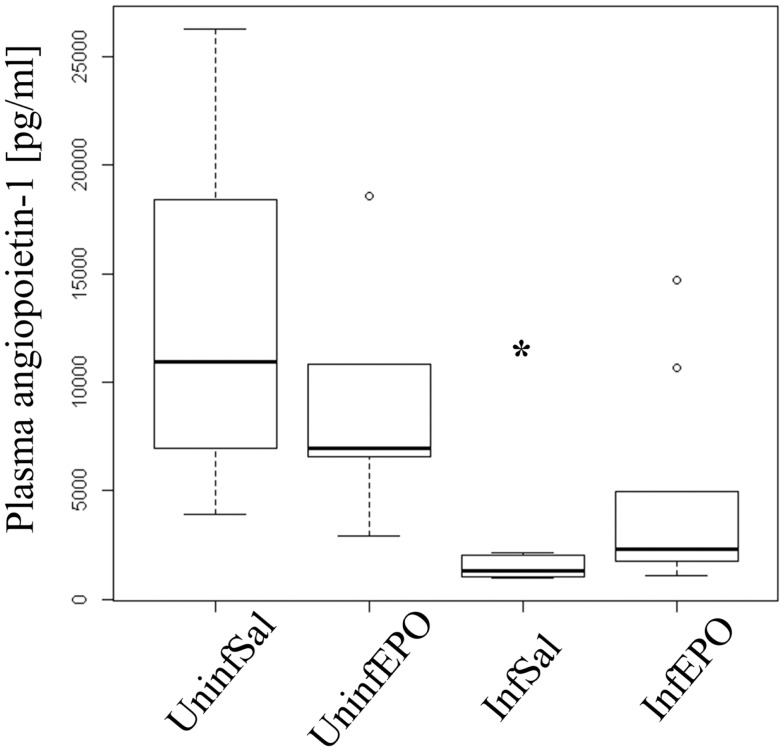
**Plasma angiopoietin-1 levels are affected by infection and EPO treatment**. In terminal CM, plasma angiopoietin-1 levels were significantly decreased (*p* = 0.004). The, InfEPO group also had lower levels of this cytokine but was statistically similar to uninfected mice (*p* = 0.06). Box plots show median values and interquartile ranges. Whiskers show Tukey hinges; open circles are outliers. Asterisks denote significant deviations from uninfected, saline-treated mice.

High levels of angiopoietin-1 are associated with endothelial stability (35) and aberrant angiopoietin-1 and angiopoietin-2 levels have been proposed as good biomarkers for severe malaria (36). Statistical analyses showed different levels of angiopoietin-1 in the experimental groups (*p* = 0.001, Figure 5) with marked reduction of angiopoietin-1 levels in terminally ill InfSal mice (*p* = 0.004). InfEPO mice also tended to have lower angiopoietin-1 levels although this was not significantly different from uninfected mice (*p* = 0.06).

## Discussion

Murine and human CM are thought to be the result of a multifaceted pathogenesis. Besides improving survival (14, 15), EPO has been shown to dampen several aspects of the pathology in murine CM including hypoxia (5), inflammation (14, 15, 37), and neuropathology (38). *P. berghei* ANKA-infected C57BL/6 or CBA mice is the most widely used model for murine CM (2, 39). It has been claimed that this model is a poor replicate of human CM (40) and indeed *P. berghei* ANKA does not export the same variant surface antigens to the erythrocyte surface as in *Plasmodium falciparum* malaria (2) and studies focussed on cerebral sequestration can not be performed using the rodent model. However, most immunological aspects as well as cerebral hypoperfusion and neuropathology have important similarities (2, 5, 39) making the murine model useful for studying human CM pathogenesis.

Here, we show systemic and cerebral increase in VEGF levels in terminally ill CM mice and show that EPO treatment reduces VEGF levels in the brain. VEGF has several opposing roles: it stimulates the growth of endothelial cells and acts neuroprotectively but also increases the permeability of the BBB (20, 41, 42). Recently, increased plasma levels of VEGF was shown to be responsible for acute lung injury in another murine model of malaria (28), but VEGF signaling has not been addressed in murine CM. Similar to our previous studies (5), we noticed increased levels of cerebral HIF-1α in CM mice. This stimulates VEGF expression in the brain (19), in line with our findings in terminally ill CM mice. HIF-1α was not increased in InfEPO mice and consequently VEGF remained normal in this group. Both hypoxia and inflammation induce VEGF expression, and since both are thought to be part of CM pathogenesis, it is difficult to determine the main inducer of cerebral VEGF expression in our model. Since EPO treatment decreases both cerebral hypoxia (5) and neuroinflammation (14, 15, 37), the study can not discriminate between the two driving forces for VEGF expression, but conclude that decreased HIF-1α and VEGF expression is associated with markedly improved clinical outcome of CM. In this study, EPO treatment also reduced parasitemia and may thus introduce bias. However, in previous works (14, 15) the effect of EPO was prominent without changed parasitemia.

Interestingly, VEGF can increase the levels of the calcium-dependent proteases, the calpains (43, 44), that break down the cytoskeleton and lead to vascular reorganization. Increased calpain levels have previously been reported in human and murine CM (45, 46). We corroborate these findings as we also found this when blotting for two independent markers of calpain activity: p25 and a 150 kDa fragment of α-spectrin. Thus, aberrant VEGF signaling may activate calpains causing endothelial pathology and neuropathology (38, 45). We found that EPO therapy reduces cerebral VEGF and markers associated with calpain activity to levels comparable with uninfected mice. However, at present we do not know whether directly blocking VEGF signaling would decrease calpain activity as well. Since plasma VEGF is significantly increased in InfEPO mice this does not seem plausible in this experimental model. Calpain activity can be induced by both hypoxia and inflammatory conditions (47, 48) and thus blocking this pathway directly may be more promising as adjunct therapy against CM.

We also corroborate our previous finding of cerebral apoptosis (14). Although apoptosis could not be detected at the mRNA level for caspase-3, histological TUNEL staining detecting DNA fragmentation has clearly demonstrated apoptosis in murine CM (14, 15). Here, we detected α-spectrin cleavage in brain homogenates to quantify the effect of caspase-3 activity and similar to previous findings, EPO treatment reduced cerebral apoptosis to levels similar to uninfected mice.

Cerebral hypoxia leads to the stabilization of HIF-1α and HIF-2α (49–51). We only detected a significant increase in HIF-1α and its downstream regulated protein VEGF, whereas cerebral HIF-2α and EPO levels remained unchanged. Others have shown an increase of cerebral EPO at the mRNA level (15), suggesting HIF-2α stabilization. However, the use of different mouse strains may contribute to this discrepancy.

Finding reliable plasma markers that can be associated with clinical severity and outcome is of interest for the management of malaria (24, 27, 52). We studied VEGF and the two receptors responsible for VEGF signaling: Flt-1 and Flk-1 (53) as well as angiopoietin-1. Flk-1 is the main receptor for VEGF-induced signaling while Flt-1 primarily acts as a decoy receptor to quench excessive VEGF; mainly in a soluble, truncated form (sFlt-1) (54). Plasma VEGF was significantly increased in both the InfSal and InfEPO groups and thus not associated with outcome and neuropathology. sFlt-1 remained unchanged in all groups. The drop in sFlk-1 in infected mice was unexpected but may likely promote pro-angiogenic signaling due to increased VEGF bioavailability and in turn improve tissue oxygenation. The sFlt-1/sFlk-1 ratio however, was only significantly changed in mice with clinical CM. These changes in VEGF and soluble receptor levels could contribute to BBB impairment due to VEGF bioavailability (28, 55). Hence, EPO seemed to act differently on the cerebral than the systemic regulation of VEGF since plasma VEGF was increased in EPO-treated malaria mice. Intra-cerebral infusions of VEGF in mice has shown that low doses induce neuroinflammation, recruitment of monocytes, and increased BBB permeability with no noticeable effect on the endothelial proliferation (56). Thus, intra-cerebral signaling may promote local neuropathology not associated with circulating levels of VEGF.

The BBB has received considerable interest in relation to CM pathogenesis. It forms a selectively permeable barrier between the central nervous system and the periphery. It also serves as anchoring point for platelets, leukocytes and infected erythrocytes perturbing the microcirculation in CM (2, 6, 57–59). Another arm of angiogenesis is the angiopoietin-Tie-2 pathway. Angiopoietin-1 and -2 signal via the Tie-2 receptor and are reliable markers of endothelial activation (24, 27). They are also predictable biomarkers of malaria severity (60, 61). Angiopoietin-1 upkeeps endothelial stability, while angiopoietin-2 stimulates endothelial remodeling. We measured a significant drop in angiopoietin-1 in murine CM, which suggests considerable endothelial activation and instability. Interestingly, InfEPO mice also had very low levels of angiopoietin-1 suggesting that endothelial function may be perturbed in these mice as well. A recent study of murine CM showed that EPO therapy decreased BBB permeability and endothelial inflammation (37), suggesting that the barrier function is still preserved in EPO-treated mice. However, in that study (37), mice were treated with EPO at day 2–4 p.i., hampering direct comparison with our study. It could be hypothesized that the endothelium is affected and activated in InfEPO mice, yet maintaining its integrity and keeping the BBB selectively permeable despite the increased VEGF and decreased angiopoietin-1 levels in plasma. CM has been termed by some as a vasculopathy (62), and studying the effects of EPO in mice without cerebral, endothelial EPO receptors (63) would be highly relevant for assessing the contributions from endothelium to CM pathogenesis.

In conclusion, these data show highly upregulated VEGF signaling in terminal, murine CM. Increased cerebral VEGF may directly contribute to neuropathology by promoting monocyte extravasation (56), BBB disruption, and calpain activity. Calpain activity is also increased in human CM (45) and inhibition of this pathway should be studied further. EPO reduces both hypoxia (64) and inflammation in murine CM (14, 15, 37), which likely prevents the upregulation of VEGF signaling pathway in the brain. Plasma VEGF was increased significantly in InfEPO mice and angiopoietin-1 levels were also decreased though insignificantly. These findings point toward EPO having a prominent role in neuroprotection in murine CM while a more modest role on the systemic levels of potential biomarkers of disease severity.

## Author Contributions

Casper Hempel designed the experiment, carried out animal studies, performed the western blots, drafted the manuscript. Nils Hoyer performed ELISA experiments of plasma samples and analyses. Anna Kildemoes performed ELISA experiments of plasma and brain samples. Charlotte Bille Jendresen carried out animal studies and prepared brain homogenates. Jørgen Anders Lindholm Kurtzhals contributed to the design of experiments. All authors critically revised the manuscript and approved the final version for publication.

## Conflict of Interest Statement

The authors declare that the research was conducted in the absence of any commercial or financial relationships that could be construed as a potential conflict of interest.
